# Biological age in healthy elderly predicts aging-related diseases including dementia

**DOI:** 10.1038/s41598-021-95425-5

**Published:** 2021-08-05

**Authors:** Julia W. Wu, Amber Yaqub, Yuan Ma, Wouter Koudstaal, Albert Hofman, M. Arfan Ikram, Mohsen Ghanbari, Jaap Goudsmit

**Affiliations:** 1 Department of Epidemiology, Harvard T.H. Chan School of Public Health, Boston, USA; 2 Department of Epidemiology, Erasmus MC University Medical Center, Rotterdam, The Netherlands; 3 Department of Immunology & Infectious Diseases, Harvard T. H. Chan School of Public Health, Boston, USA; 4 Human Vaccines Project, New York, NY USA

**Keywords:** Biomarkers, Medical research

## Abstract

Application of biological age as a measure of an individual´s health status offers new perspectives into extension of both lifespan and healthspan. While algorithms predicting mortality and most aging-related morbidities have been reported, the major shortcoming has been an inability to predict dementia. We present a community-based cohort study of 1930 participants with a mean age of 72 years and a follow-up period of over 7 years, using two variants of a phenotypic blood-based algorithm that either excludes (BioAge1) or includes (BioAge2) neurofilament light chain (NfL) as a neurodegenerative marker. BioAge1 and BioAge2 predict dementia equally well, as well as lifespan and healthspan. Each one-year increase in BioAge1/2 was associated with 11% elevated risk (HR 1.11; 95%CI 1.08–1.14) of mortality and 7% elevated risk (HR 1.07; 95%CI 1.05–1.09) of first morbidities. We additionally tested the association of microRNAs with age and identified 263 microRNAs significantly associated with biological and chronological age alike. Top differentially expressed microRNAs based on biological age had a higher significance level than those based on chronological age, suggesting that biological age captures aspects of aging signals at the epigenetic level. We conclude that accelerated biological age for a given age is a predictor of major age-related morbidity, including dementia, among healthy elderly.

## Introduction

Prevention of aging-related diseases (ARDs) is paramount in the current era of population aging. An improved understanding of the aging process can potentially extend healthspan and reduce ARDs burden. It has long been observed that the pace of aging varies from person to person. This highlights a key concept that due to underlying biological mechanisms, biological age at an individual level can be separated from chronological age^1–3^. As opposed to chronological aging, an index of the passage of human time, biological aging refers to the underlying aging processes at the biological level. More specifically, it concerns with the underlying, disease-independent accumulation of pathophysiological changes that contribute toward mortality over time^1–3^. Biological age, defined by clinical and molecular biomarkers, indeed predicts overall mortality and ARDs, sometimes even better than chronological age^4^. Modifiable risk factors as a whole predict mortality reasonably well over time, even though independent genetic factors predict mortality only modestly^5^. Investigating biological age can help identify individuals at higher risk of disease and death, before clinical manifestation of disease. Biomarkers of biological age have great potential in evaluating healthy-aging intervention programs, selecting suitable candidates for clinical trials, as well as indicating levels of personal health, and predicting risk of aging-related diseases. These applications may provide insight on how to extend both lifespan and healthspan and cope with the burden of ARDs worldwide.

The concept of biological age has been constructed and validated in large human cohort studies for a panel of physiological biomarkers^6–8^, differentially methylated sites in DNA (DNAm age)^9,10^, circulatory metabolites (metabo-age), the levels of messenger-RNAs^11^ and microRNAs (miRNAs) in both whole blood^12^ and plasma samples (Wu J.W. et al., manuscript submitted). Physiological biomarkers tend to be stronger predictors of mortality and aging-related morbidity outcomes than molecular biological age measures^4^, indicating the complexity of human aging processes. Assessments of biological age can provide an overview of one’s general physiological health (as measured by glucose, mean red cell volume, white blood cell count, C-reactive protein among other markers)^21^. Furthermore, physiological health is closely related to neurological health, of which the mechanisms underlying brain aging are poorly understood^22^. Studying neurological aging in conjunction with physiological aging is meaningful, given that substantial neurodegeneration has already occurred when cognitive decline and dementia manifest^23^. Therefore, a panel of biological age markers may allow us to detect mechanisms underlying both physiological aging processes and brain aging process, so that targets of intervention can be identified and ultimately be optimized for prevention.

Most of the biological age measures rely on the association between composite biomarkers and chronological age might be imperfect because of the practice of minimizing the deviation from chronological age through regression and not estimating this entity of interest empirically^4^. The main knowledge gap is to what extent the deviation of biological age measure from chronological age predicts risks of mortality and major ARDs onsets, especially dementia, in advanced age populations. We sought to ask the following question: is it possible to build a physiological marker-based biological age algorithm that predicts elevated risk of all major aging-related diseases including dementia for a given chronological age?

In a previous study^13^, we have validated the approach of Klemera and Doubal^14^ and showed that over a median follow-up of 11 years, biological age at baseline was superior to chronological age and traditional biomarkers, in predicting mortality, morbidity and onset of specific diseases such as stroke and cancer. Furthermore, compared to chronological age alone or combined with the individual biomarkers, adding a brain biomarker for neurological degeneration (plasma NfL, total-tau, amyloid beta-40 and -42) further improved the association of biological age with dementia, including Alzheimer disease (AD). On the other hand, the Klemera and Doubal-based biological age did not predict mortality or morbidity when adjusting for chronological age, and had stronger associations with chronological age than with risk of dementia. It became evident that a completely new model for biological age, beyond chronological age, was needed to improve both the association with prediction of mortality and overall morbidity, including dementia.

In this study, we aimed to improve biological age algorithms through a population-risk-based framework. Recent work by Levine et al.^4^, has introduced a novel measure of ‘phenotypic age’, developed and validated on NHANES III (n = 9926). A Gompertz proportional hazards regression^14^ was applied to account for the hazard of mortality when selecting clinical biomarkers in the training dataset. This approach is unlike most of the previous biological age algorithms, which largely aimed to model chronological age as the dependent variable. As a result, it is able to capture the association between accelerated biological age and elevated risk of age-related comorbidity. However, Levine’s “phenotypic age” algorithm was unable to establish a significant link between accelerated aging and elevated risk for dementia. We hypothesized that this caveat was not due to the population-risk-framework itself, but because the “phenotypic age” was trained in the NHANES data, which has a relatively younger age distribution, than is common for neurological outcomes. Furthermore, we investigated whether by including markers of neurodegeneration, we could improve the prediction of neurological outcomes in advanced-aged cohorts.

Our approach to improve the biological age models comprised of two steps. First, we validated Levine’s “phenotypic age” algorithm in the Rotterdam Study (n = 1930). Second, we developed and validated new biological age algorithms in the Rotterdam Study using the same Gompertz proportional hazards regression framework plus neurodegenerative markers (NfL, total-tau, amyloid beta-40 and -42). MicroRNAs (miRNAs) age signature was found to be not only predictive of the actual age, but useful as a biomarker of all-cause mortality in both whole blood^12^ and plasma samples (Wu J.W. et al., manuscript submitted). We assessed the deviation of biological age from chronological age through the plasma miRNA expression signature.

## Results

### Development of BioAge1 and BioAge2 algorithms

This study included a subset of participants from the Rotterdam Study, a prospective community-based cohort study (see Fig. S1). In total 1930 participants from RS-I and RS-II were followed-up between 2002 and 2012, with a mean follow-up time of 7.2 (SD = 1.8) years, with ascertainment of 411 all-cause mortalities. The mean age for this study sample was 71.6 years (SD = 7.6) (see Table 1 for baseline characteristics), representing an advanced age population. We fitted Gompertz proportional hazard models on a training subset (n = 1158), and developed two new biological age algorithms: one including the same set of ten predictors as in the “phenotypic age” algorithm (BioAge1, summarized in Table 2) and the other including brain marker NfL in addition to the set of ten predictors (BioAge2, summarized in Table 3). We also explored biological age algorithms including either total-tau, amyloid beta-40, or -42, but none of these brain markers were significant.

**Table 1:** Descriptive characteristics of the study population (n = 1930).

Characteristics	Study population (n = 1930)
Age, mean (SD)	71.6 (7.6)
Female (%)	1099 (56.9%)
**Smoking status**	
Current (%)	280 (14.5%)
Former (%)	1072 (55.5%)
Never (%)	578 (29.9%)
BMI (kg/m2), mean (SD)	27.6(4.1)
Total cholesterol, mean (SD)	5.6(1.0)
High-density lipoprotein, mean (SD)	1.5(0.4)
Glucose (mmol/L), mean (SD)	5.8(1.4)
Systolic blood pressure (mmHg), mean (SD)	148.1 ± 21.0
Diastolic blood pressure (mmHg), mean (SD)	79.5 ± 10.9
Prevalent type 2 diabetes (%)	264 (13.7%)
Prevalent stroke (%)	79 (4.1%)
Prevalent coronary heart disease (%)	210 (10.9%)
Prevalent dementia (%)	11 (0.6%)
Prevalent chronic obstructive pulmonary disease (%)	171 (8.9%)
Prevalent any cancer (%)	294 (15.2%)
Incidence type 2 diabetes	153
Incidence stroke	82
Incidence coronary heart disease	139
Incidence dementia	167
Incidence chronic obstructive pulmonary disease	165
Incidence any cancer	315

**Table 2:** Gompertz coefficients developed in the Rotterdam study for BioAge1: without brain markers.

Variable	Marker of	Units	Weight
Albumin	Liver	g/L	− 0.043
Creatinine	Kidney	umol/L	0.013
Glucose, serum	Metabolic	mmol/L	0.153
C-reactive protein (log)	Inflammation	mg/dL	0.015
Lymphocyte percent	Immune	%	− 0.017
Mean (red) cell volume	Immune	fL	0.035
Red cell distribution width	Immune	%	0.083
Alkaline phosphatase	Liver	U/L	0.008
White Blood Cell count	Immune	1000 cells/uL	0.072
Age		Years	0.089

**Table 3:** Gompertz coefficients developed in the Rotterdam study for BioAge2: with brain marker NfL.

Variable	Marker of	Units	Weight
Albumin	Liver	g/L	− 0.044
Creatinine	Kidney	umol/L	0.009
Glucose, serum	Metabolic	mmol/L	0.150
C-reactive protein (log)	Inflammation	mg/dL	0.006
Lymphocyte percent	Immune	%	− 0.017
Mean (red) cell volume	Immune	fL	0.034
Red cell distribution width	Immune	%	0.059
Alkaline phosphatase	Liver	U/L	0.007
White Blood Cell count	Immune	1000 cells/uL	0.074
Neurofilament level (log)	Brain	pg/ml	0.529
Age		Years	0.071

### BioAge1 and BioAge2 predict risk of all-cause mortality

In the validation subset (n = 720 with non-missing covariates) of the Rotterdam study data, we validated the two new biological age algorithms: BioAge1 and BioAge2. BioAge1 was highly correlated with chronological age (r = 0.85) and PhenoAge (r = 0.96). Table 4 shows the association between BioAge1 and risk of all-cause mortality, based on Cox proportional hazard models in this validation subset. Each one-year increase in BioAge1 was associated with 11% (HR 1.11; 95%CI 1.08–1.14; p < 0.0001) elevated risk of mortality, after adjusting for chronological age, APOE status and gender. When restricting the outcome within the first 5-year and 3-year time intervals, each one-year increase in BioAge1 was associated with 15% and 16% elevated risks of mortality respectively, after adjusting for chronological age, APOE status and gender. Similarly, BioAge2 was highly correlated with chronological age (r = 0.85) and PhenoAge (r = 0.95). Table 5 shows the association between BioAge2 and risk of all-cause mortality, based on Cox proportional hazard models in this validation subset. Each one-year increase in BioAge2 was associated with 11% (HR 1.11; 95%CI 1.08–1.14; p < 0.0001) elevated risk of mortality, after adjusting for chronological age, APOE status and gender. When restricting the outcome within the first 5-year and 3-year time intervals, each one-year increase in BioAge2 was associated with 14% and 16% elevated risks of mortality respectively, after adjusting for chronological age, APOE status and gender. Even though chronological age alone strongly predicted mortality in univariate models, in all final adjusted models chronological age became no longer significant for predicting mortality for a given biological age (see Tables 4 and 5).

**Table 4:** Association of BioAge1, 3-Year, 5-Year and total all-cause Mortality: The Rotterdam Study, 2002–2012.

Variables	3-year Mortality				5-year Mortality				Total Mortality			
	Univariate		Final model		Univariate		Final model		Univariate		Final model	
	HR	P	HR	P	HR	P	HR	P	HR	P	HR	P
Age	1.13	< .0001	0.95	0.156	1.14	< .0001	0.99	0.555	1.13	< 0.0001	1.02	0.368
Female	0.49	0.020	0.89	0.746	0.59	0.017	1.21	0.455	0.64	0.004	1.10	0.594
APO E	0.83	0.577	0.75	0.398	0.85	0.522	0.81	0.392	1.03	0.846	0.99	0.968
BioAge1	1.13	< 0.0001	1.16	< 0.0001	1.14	< 0.0001	1.15	< 0.0001	1.12	< 0.0001	1.11	< 0.0001

**Table 5:** Association of BioAge2, 3-Year, 5-Year and total all-cause Mortality: The Rotterdam Study, 2002–2012.

Variables	3-year Mortality				5-year Mortality				Total Mortality			
	Univariate		Final model		Univariate		Final model		Univariate		Final Model	
	HR	P	HR	P	HR	P	HR	P	HR	P	HR	P
Age	1.13	< .0001	0.96	0.193	1.14	< .0001	0.99	0.816	1.13	< 0.0001	1.02	0.198
Female	0.49	0.020	0.85	0.656	0.59	0.017	1.13	0.630	0.64	0.004	1.04	0.838
APO E	0.83	0.577	0.73	0.357	0.85	0.522	0.78	0.324	1.03	0.846	0.97	0.840
BioAge2	1.13	< 0.0001	1.16	< 0.0001	1.14	< 0.0001	1.14	< .0001	1.12	< 0.0001	1.11	< 0.0001

### BioAge1 and BioAge2 algorithms predict risks of aging-related morbidities including dementia

**Figure 1:**
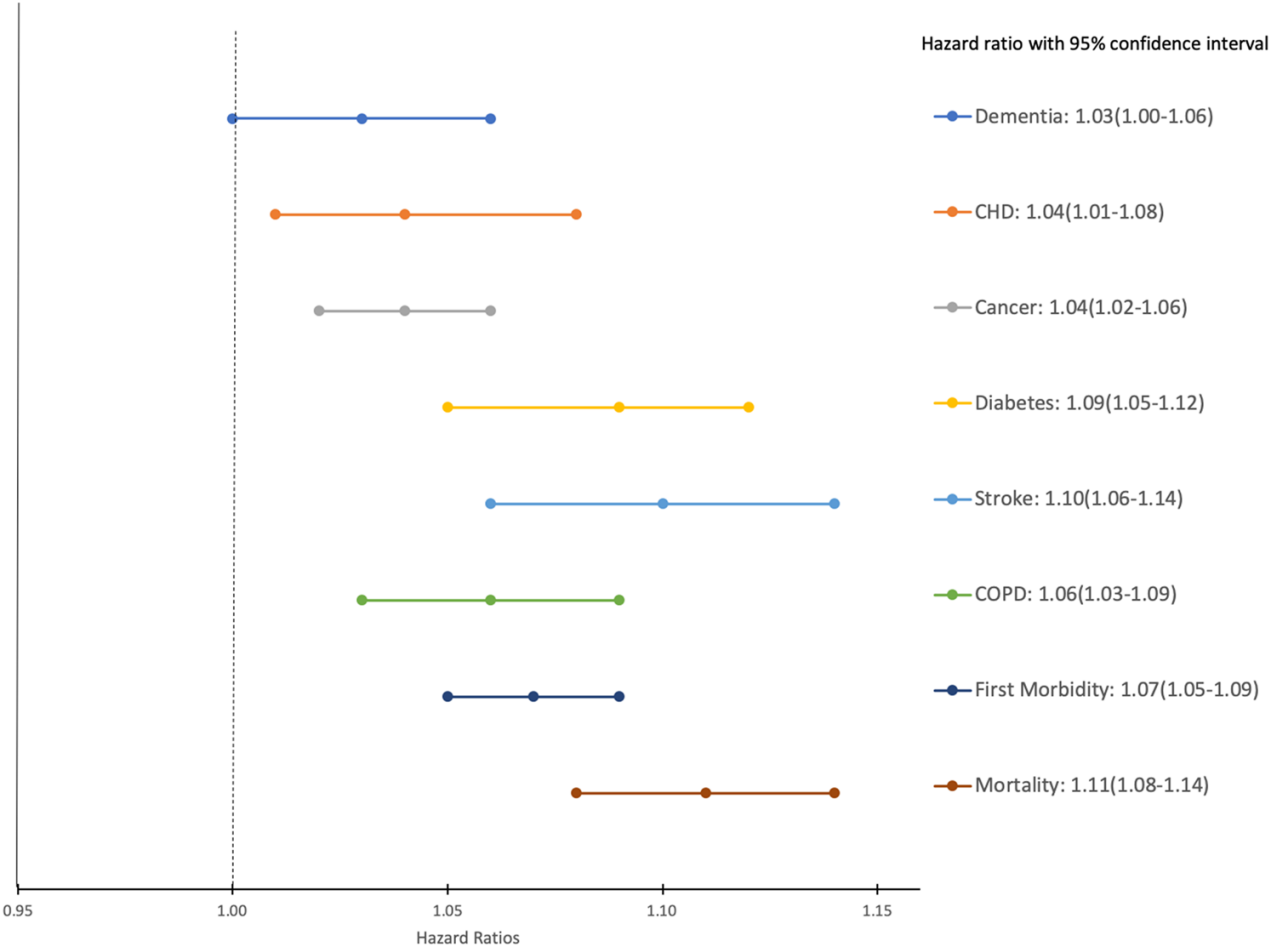
Association of BioAge1 and risk of morbidities /mortality, with adjustment for chronological age, APOE*-ε*4 and gender, the Rotterdam Study, 2002–2012.

We explored whether BioAge1 and BioAge2 algorithms predict additional risks of aging-related morbidities/ARDs, for people with fixed chronological age. Figure 1 shows the association between BioAge1 and risk of aging outcomes (BioAge2 yielded similar results; see Fig. S2). Each additional year increase in BioAge1 was associated with 7% elevated risk of first morbidities, after adjusting for chronological age, APOE status and gender. Note that each one-year increase in BioAge1 and BioAge2 was associated with 3% (HR 1.03; 95%CI 1.00–1.06; p = 0.08) and 5% (HR 1.05; 95%CI 1.02–1.08; p = 0.001) elevated risk of dementia, respectively, after adjusting for chronological age, APOE status and gender. PhenoAge was associated with 2% elevated risk of dementia (HR 1.02; 95%CI 0.99–1.04; p = 0.24) for the same adjustment with lower significant level.

### Plasma-based microRNA aging signature: biological age vs. chronological age

**Figure 2:**
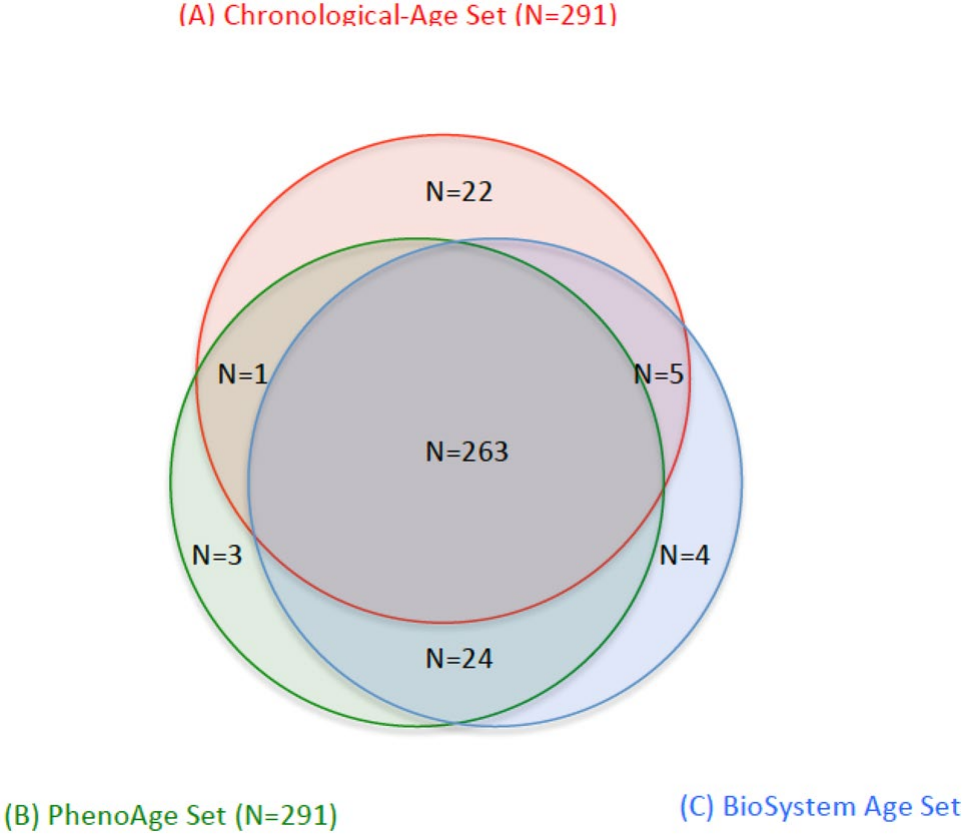
Venn Diagram showing miRNA sets that are significantly (p-value less than the Bonferroni adjusted cut-off of 0.000085) associated with (**A**) Chronological Age (n = 291, in red); (**B**) PhenoAge (n = 291, in green); (**C**) BioAge1 (n = 296, in blue).

**Figure 3:**
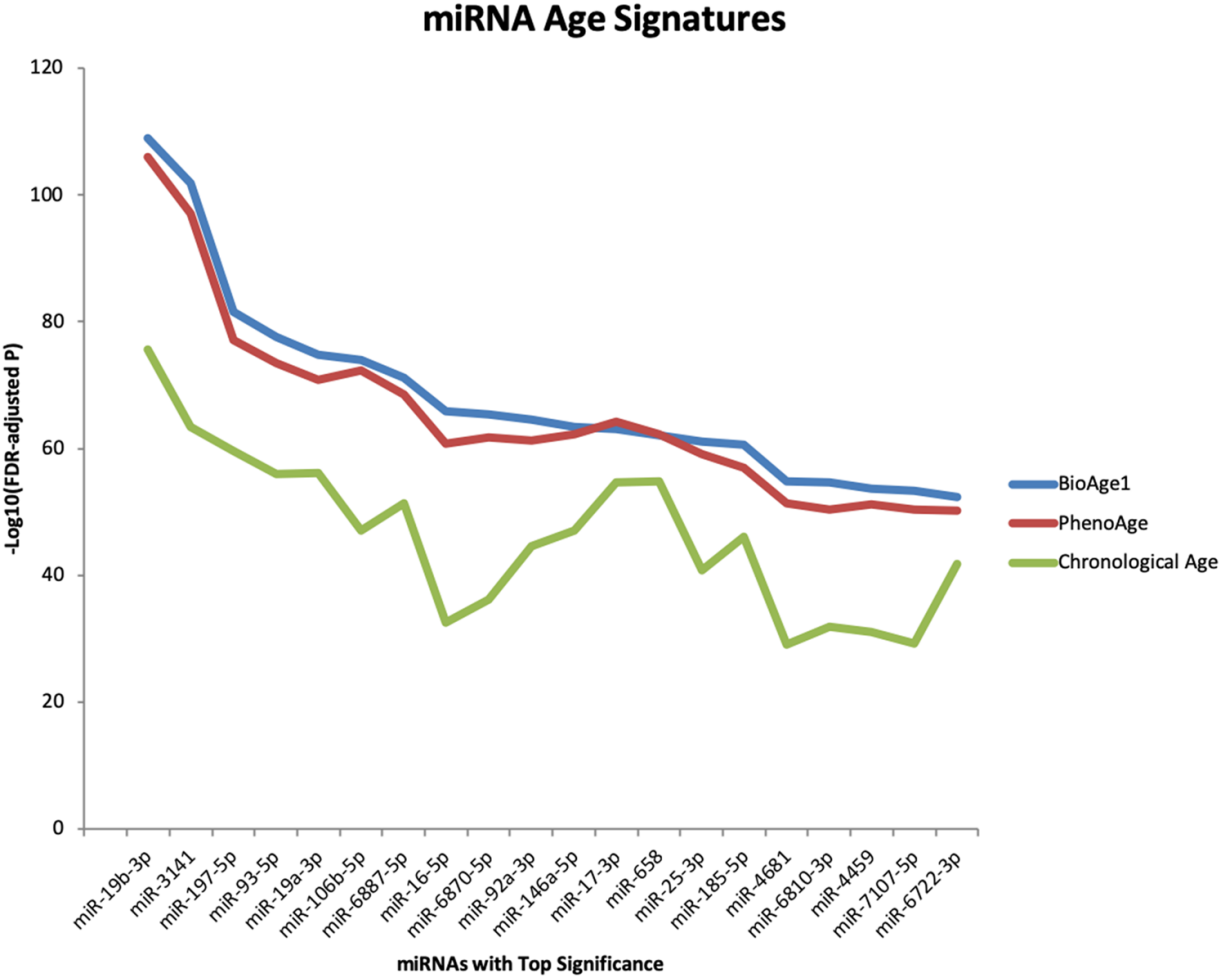
MicroRNA aging signature: comparing biological age vs. chronological age. X-axis: miRNAs that were most significantly associated with BioAge1 with gender adjustment; Y-axis: the associated adjusted p-values at the − Log10 scale, when the association was assessed for BioAge1 (blue), PhenoAge (red) and Chronological Age (green).

DNA methylation-based biological age (DNAm age) has been widely recognized as an epigenetic clock. In addition to DNA methylation and histone modifications, miRNAs have been classified as important epigenetic markers. Using the more robust quantification of RNA-seq we determined expression levels of 2083 plasma miRNAs. Of these, 591 miRNAs remained as well-expressed after normalization. We identified 291 miRNAs that were differentially expressed in relation to chronological age, at the Bonferroni-corrected p < 8.5 × 10^–5^ (0.05/591 well-expressed miRNAs), Table S1 summarizes the top 20 miRNAs among them. Similarly, by using biological age, specifically BioAge1 and PhenoAge, we also identified 296 and 291 miRNAs respectively that were differentially expressed. Tables S2 and S3 summarize the top 20 miRNAs in relation to BioAge1 and PhenoAge. These three sets of significant miRNAs were highly overlapping (Fig. 2), with 263 miRNAs presented in all three sets, indicating the similarity of the miRNA aging signatures in relation to biological age vs. chronological age. In comparison to models including just chronological age, the top differentially expressed miRNAs had much higher significance levels (Fig. 3) in biological age models, based on the much lower FDR-adjusted p-values (see Table S3) vs. those based on chronological age (see Table S2), also summarized in Fig. 3 at -log10 scale. This suggests that biological age models (or algorithms) are capable of condensing aging signals and predicting mortality for people of a fixed chronological age.

## Discussion

We explored the application of Gompertz proportional hazard regression^15^, as a platform integrating multiple biological system data accounting for every person’s observed mortality risk. We developed and validated two new biological age algorithms in the advanced age Rotterdam Study (n = 1930). The measure of the biological age itself involved chronological age as a variable, and thus was not independent of chronological age. Our study aimed to examine the predictive value of biological cage that is beyond and above chronological age. We found that biological age was associated with mortality risk, after adjustment of chronological age, APOE status and gender status. In other words, for a fixed chronological age, APOE status and gender, increase of biological age was found to be associated with elevated risk of all-cause mortality as well as major ARDs such as coronary heart disease, diabetes, cancer, stroke, COPD and dementia. These findings support our hypothesis of the additive predictive value of biological age. To further investigate the deviation of biological age from chronological age, we compared the miRNA expression signatures based on biological age and chronological age.

Of the various methodologies deployed for biological age development, most were based on the association between composite biomarkers and chronological age, which did not account for the deviation from chronological age, the exact entity that biological age was expected to capture. In 2018, Levine et al. proposed a revolutionary approach of incorporating observed mortality risk using large cohort data. In their model, the biological age reflected the putative age in the general population that corresponds to the risk of mortality^4^. Using this innovative modeling, the authors developed and validated a phenotypic age (PhenoAge) construct in NHANES cohorts to predict all-cause mortality and all major aging-related morbidity, with the exception of cerebrovascular disease and dementia. Here we confirmed our hypothesis that the caveat was not due to the population-risk-approach or biological age itself, but because the “phenotypic age” was trained in the NHANES data with a relative younger age structure than common for neurological outcomes. Biological age algorithms trained in advanced age cohorts indeed predict the elevated risk for all major aging-related morbidity, including dementia, associated with accelerated aging.

In addition to biological age algorithms, several frailty indices have been developed to identify individuals with multi-system comorbidities and high risk of mortality^24^. The major shortcoming of these frailty indices is their ceiling effect at high age groups, which indicates that there is a greater level of unmeasured heterogeneity of frailty in advanced ages^25^. This is in line with a previous study among elderly Canadians, that reported a wider spread of biological age in a population older than 77 years, whereas the frailty index leveled off^26^. The components required to calculate a frailty index may also be time-consuming and may require clinical expertise, especially when neuropsychological testing and motor function tests are involved. Conversely, the biomarkers in our biological age algorithms are easy to measure and interpret. Not only do these biomarkers reflect upper-stream processes underlying age-related diseases, some of them (albumin, creatinine, alkaline phosphatase) are used in clinical settings for patients with multi-system organ failure to predict mortality^27,28^.

We investigated whether we could improve the prediction of the biological age model, particularly for dementia and stroke, by including markers of neurodegeneration. We have incorporated NfL, the only significant neurodegenerative marker in our study, into our novel BioAge2 algorithm. Previous studies^16–18^ as well our own analysis of a longitudinal study^19^ strongly indicate that NfL, a relatively unspecific marker of neuroaxonal damage, was the best predictor of neurological outcomes. Our findings show that incorporating NfL into the biological age algorithm further improved the prediction of the elevated risk for dementia, although the added value was not impressively large, relative to the high cost of measuring NfL. Plasma NfL alone (after adjustment of age, gender and APOE-ε4) was also predictive of dementia. However, when BioAge1 was included in the prediction model, the additional predictive gain was small. As a matter of fact, NfL was highly correlated with BioAge1. Its small incremental gain was possibly because the nine blood markers had already largely explained the variance that NfL otherwise would have explained. Indeed, in our validation set our newly developed BioAge1 and BioAge2 were highly correlated with NfL. We therefore focused on BioAge1 and not BioAge2 for further evaluations.

The miRNA analyses confirmed the deviation of biological age from chronological age since while 263 miRNAs were significantly associated with biological and chronological age alike, the top 20 differentially expressed miRNAs in biological age had a much higher significance level than those in chronological age. Among the top significant ones we identified, miR-19b, miR-19a, miR-92a and miR-17 were all members of the miR-17–92 cluster, with crucial function in stem cell cycle regulation for supporting cellular reprogramming^29^, indicating a potential link of the miR-17–92 cluster involvement in the plasma miRNA aging mechanism. Similarly, miR-106b that shares sequence homology with miR-17–92 cluster members, is involved in cell proliferation by targeting p21, a protein important in the cell-cycle regulation^30^. We also found miR-106b-5p among the top significant list, suggesting its potential role in aging. These findings suggest that biological age algorithms likely have captured aging signals at the cell-cycle regulation level. In addition, even though chronological age alone strongly predicts risk for mortality, in all final adjusted models chronological age became no longer significant for predicting mortality for a given biological age, further confirming a superior predictive capacity of biological age to that of chronological age.

The finding that the new biological age algorithms were predictive of dementia including AD has important implications. Accelerated biological age indeed predicts an elevated risk of mortality and all major age-related morbidities including dementia outcomes for people of a fixed chronological age, supporting the key concept of biological age and its important role in the field of healthy aging. Dementia and Alzheimer’s disease pathology have been extensively studied with domain-specific biomarkers and panels^31^. On the other hand, instead of focusing on prediction of dementia pathology, our goal is rather to explore whether physiological health-based composite markers can predict risk of multi-system aging-related outcomes including dementia. Such composite markers will provide utility at a population level for monitoring and evaluation of interventions for preventing all-across-the board aging related outcomes including dementia, that are more upper-stream than pathology-based approaches. Future work can be done to train and validate additional biological age algorithms alike based on slightly different sets of physiological biomarkers to accommodate data availability. Further studies are warranted to evaluate whether these biological age algorithms can be applied to other neurodegenerative disorders.

A major strength of our study is the usage of a large community-based advanced- age cohort study data with a novel RNA-sequencing method to assay the levels of a wide array of miRNAs in stored plasma samples. The mortality and morbidity outcomes were well-ascertained. Nevertheless, there were several limitations with our study. First, we have not used an independent cohort outside of the Rotterdam Study to replicate our results even though we have conducted validation and established internal validity. Secondly, one additional year of accelerated aging at age 70 might not be equivalently detrimental to that of age 80. However, we did not model this non-linearity as we did not want to make the model overly difficult to understand. This differentiation was not our main focus, and what we presented should be considered as an average effect across age groups. Third, although predictive models, such as these algorithms, can give us hypothesis-generating insight into underlying mechanisms, the associations do not imply causation. Therefore, to see whether components of biological age actually underlie dementia-related processes, further causal studies incorporating pathway analysis and mendelian randomization studies for example, are required. This limitation, nevertheless, will not affect the prediction-based utility aspect for surveillance and healthy-aging program evaluation. Fourth, previous studies have shown the importance of omic-based biological age such as DNAm-age and metabo-age. In this study we did not have DNA methylation, gene expression or metabolomics data available from the same time point to calculate biological age based on such omics data. However, miRNA aging signatures were identified in relation to biological age vs. chronological age. The top differentially expressed miRNAs in relation to biological age had much higher significance level, suggesting that biological age algorithms capture some aspect of aging signals at the epigenetic level other than DNA methylation.

We have shown that in a large community-based advanced-age population, accelerated biological age for a given age predicts lifespan, healthspan and risks for all major age-related morbidity including dementia. Similar plasma miRNA aging signatures were identified in relation to biological age vs. chronological age. The top differentially expressed miRNAs in relation to biological age had much higher significance level, suggesting epigenetic links with biological age algorithms beyond DNA methylation.

## Methods

### Study settings

This study included participants from the Rotterdam Study, a prospective community-based cohort study^20^. In 1990, residents aged 55 years and older residing in Ommoord, a district of Rotterdam, the Netherlands, were invited to participate in the study. Of 10,215 invited inhabitants, 7983 agreed to participate in the baseline examinations. In 2000, 3011 participants (of 4472 invitees) who had reached 55 years of age or moved into the study district since the start of the study were added to the cohort. In 2006, a further extension of the cohort was started in which 3932 participants, of 6057 invited, aged at least 45 years living in Ommoord were included. Follow-up examinations take place every 3–4 years. The Rotterdam Study was approved by the Institutional Review Board at Erasmus Medical Center, the Netherlands, and performed in accordance with the Declaration of Helsinki. For this study, 2000 individuals were randomly selected from the fourth round of Rotterdam Study-I (RS-I-4) and second round of Rotterdam Study-II (RS-II-2) between 2002 and 2005. The criteria for inclusion included the availability of informed consent and valid serum samples that were both available at the visits in the Rotterdam Study. After exclusion of 70 participants with missing or extreme values, data analyzed in this study concern 1930 participants with physiological and neurological function information available (Fig. S1). All research was performed in accordance with relevant guidelines/regulations.

### Calculation of biological age

We used the following two-fold approach for developing our biological age models: 1) We first validated Levine’s “phenotypic age” algorithm in the Rotterdam Study (n = 1930); 2) We then deployed a 60:40 split of training/validation sets on the Rotterdam study data (n = 1930) to develop and validate new biological age algorithms (BioAge1 and BioAge2) using the same Gompertz proportional hazards regression modeling framework, with or without incorporating the neurodegeneration markers (NfL, total-tau, amyloid beta-40 and -42).

### Validation of Levine’s “phenotypic age” algorithm

We first calculated biological age based on the “phenotypic age” algorithm that Levine et al. initially developed and validated based on the NHANES data ^4^. We included the parameters from Levine et al. in the supplemental material (see Table S1). All the weights and parameters were carried over from the original study and implemented in the Rotterdam study cohort data (n = 1930). This implementation was based on chronological age and 9 biomarkers (albumin, creatinine, glucose, [log] C-reactive protein (CRP), lymphocyte percent, mean cell volume, red blood cell distribution width, alkaline phosphatase, and white blood cell count) that were selected in the “phenotypic age” algorithm^4^. For scoring, we used the parametric proportional hazard model based on the Gompertz distribution as described in Levine et al., and estimated the 10-year mortality risk of the j-th individual based on the cumulative distribution function of the model:

1 $$\mathrm{MortalityScorej }=\mathrm{ CDF}(10,\mathrm{ Xj}) = 1-{e}^{-{e}^{xjb}(\mathrm{exp}\left(10*\gamma \right)-1)/\gamma }$$

where $xb$ represented the linear combination of biomarkers from the fitted model of phenotypic age. Both $b$ and $\gamma$ were estimated from the NHANES cohort data^4^. To obtain the “phenotypic age”, the mortality score was converted into units of years, based on parametrization of a separate Gompertz proportional hazard model fit using only chronological age from the NHANES ^4^.

### Development and validation of the new biological age algorithms

We then calculated biological age based on what we developed and validated using the Rotterdam study data. We employed validations with a 60:40 split of training/validation sets on the Rotterdam study cohort data (n = 1930). In the training set, we re-fit parametric proportional hazard models based on the Gompertz distribution in the Rotterdam Study cohorts, using 1) the same set of ten predictors as in the “phenotypic age” algorithm; 2) the ten predictors as in the “phenotypic age” algorithm, as well as the addition of one of the four brain markers (plasma neurofilament level NfL, total-tau, amyloid beta-40 and -42). In the validation set, we estimated the 10-year mortality risk of the j-th individual based on the cumulative distribution function of the models obtained in Eq. (1), in accordance with the procedures conducted in validating the phenotypic age algorithm but using the linear combination of biomarkers with parametrization from the newly fitted models of biological age (see Tables 3 and 4). To obtain the biological age, the mortality score was converted into units of years, based on parametrization of a separate Gompertz proportional hazard model fit using only chronological age from the Rotterdam cohort training set.

### Association between biological age and risk of all-cause mortality/morbidities

Since PhenoAge was developed from an external data set, we first validated among the full Rotterdam study cohort data to establish the modeling approach. We then separately tested it on the same validation set as BioAge1 and BioAge2 for the purpose of comparison. For validation of the PhenoAge algorithm, we first examined the correlation between PhenoAge and chronological age. Next, we used Cox proportional hazard models to assess the association between Phenotypic Age and risk of all-cause mortality, with adjustment for chronological age, APOE status and gender. Follow-up was truncated on January 1st 2012 in the Cox-models of all-cause mortality. We used three time-intervals to examine the risks of mortality: 3-year mortality, 5-year mortality and total mortality. We assessed the association between BioAge1/2 algorithms and risk of all-cause mortality similarly in the validation set. Hazard ratios were expressed as annual risk of death over the follow up period.

To assess the association between the biological age and risk of morbidities, prevalent cases of the outcome of interest were excluded from the respective analyses. The morbidities included: diabetes mellitus, stroke, CHD, dementia, COPD and cancer (any cancer and specific cancers, such as lung cancer, breast cancer and other cancers with a significant incidence during follow-up). First morbidity was defined as first occurrence of any of these major morbidities. We focused on BioAge1 and BioAge2, but also included PhenoAge in this analysis for comparison.

### Ascertainment of outcomes

The outcome measures for this analysis were all-cause mortality and morbidities. Outcome analyses included all deaths/morbidity endpoints that occurred prior to January 1st 2012. Information on vital status of participants was obtained on a weekly basis via municipal population registries and through general practitioners’ and hospitals’ databases. Events were coded according to the International Classification of Diseases 10th version (ICD-10) by two independent research physicians. All-cause mortality is defined as participants who died from any cause during the total follow-up period, which was completed until January 1st 2012.

### Dementia screening and surveillance

Ascertainment methods for dementia in the Rotterdam Study, have previously been described in detail^32^. Participants were screened for dementia using the Mini-Mental State Examination (MMSE) and the Geriatric Mental Schedule (GMS) organic level, at each center visit. Participants with MMSE < 26 or GMS > 0 were invited for further assessment and informant interview. In addition, the entire cohort was under continuous surveillance for incident dementia using computerized linkage of the study database with medical records from general practitioners and the regional institute for outpatient mental health care. If required, clinical neuroimaging data were used to determine dementia subtype. A consensus panel, including a consultant neurologist, established the final diagnosis for every participant suspect for dementia using standard criteria for dementia (DSM-III-R) and AD (National Institute of Neurological and Communicative Disorders and Stroke–Alzheimer’s Disease and Related Disorders Association). Follow-up until January 1, 2012, was near complete and participants were censored within this follow-up period at date of dementia diagnosis, date of death, date of loss to follow-up, or January 1st, 2012, whichever came first.

### MicroRNA expression profiling and normalization

Expression levels of 2083 plasma miRNAs were determined by the HTG EdgeSeq miRNA Whole Transcriptome Assay (HTG Molecular Diagnostics, Tuscon, AZ, USA) and the Illumina NextSeq 500 sequencer (Illumina, San Diego, CA, USA). MiRNA specific probes were hybridized to each miRNA.

The bioinformatics workflow consisted of two parallel paths, one for the quality control (QC) checks and one for actual results processing. In both cases we used in-house scripts. For the QC checks, first the average amount of ANT probe signal was tested relative to the total signal of the sample. If the relative ANT probe signal was too high, a sign that the signal of the sample was too low, the sample was either re-tested (if re-test material was available) or otherwise rejected. For the actual results processing, we used counts per million (CPM) to quantify miRNA expression. For the purpose of standardization of adjustment for total reads per sample, Log2 transformation of CPM was used. We then applied a cut-off of 1.0 so that Log2 CPM < 1.0 were considered as non-expression in the samples. Finally, we used the lower limit of quantification (LLOQ) method for normalization to select well-expressed miRNAs, by modelling the relation between mean and standard deviation of Log2 CPM among the 1930 participants with a monotonic smooth spline fit. The miRNAs with 50% expression values above LLOQ were defined as well-expressed in plasma. Out of the 2083 measured miRNAs, 591 miRNAs were considered well-expressed. More details were described in a previous study^33^.

### Identifying differentially expressed miRNAs in relation to biological age

To identify age-related miRNA expression in plasma, we used linear regression to model individual miRNA expression as the dependent variable and biological age as an explanatory variable, with adjustment of gender. We computed false discovery rate (FDR) to account for multiple testing. We constructed a volcano plot (with − log10(pvalue) on the y-axis and fold change per 40 years on the x-axis) to display the significance and magnitude of each bivariate association. We then compared the top biological age-related miRNAs to those identified in relation to chronological age.

## Supplementary Information

Supplementary Information.

## Data Availability

For miRNA and clinical data access, please contact MG at m.ghanbari@erasmusmc.nl.
