# Supplementary material for: Biological age in healthy elderly predicts aging-related diseases including dementia
Source: Sci Rep. 2021 Aug 5;11:15929. doi: 10.1038/s41598-021-95425-5 (PMC8342513; doi:10.1038/s41598-021-95425-5)
Supplement: Supplementary file 1 — Supplementary Information. [file 41598_2021_95425_MOESM1_ESM.docx]

**Biological age in healthy elderly predicts dementia in the absence of a brain marker**

Julia W. Wu^1,2*^, Amber Yaqub^2^, Yuan Ma^1,2^, Wouter Koudstaal^4,^ Albert Hofman^1,2^, M. Arfan Ikram^2^, Mohsen Ghanbari^2#^, Jaap Goudsmit^1,3#^

^1^Department of Epidemiology, Harvard T.H. Chan School of Public Health, Boston, USA

^2^Department of Epidemiology, Erasmus MC University Medical Center, Rotterdam, The Netherlands

^3^Department of Immunology & Infectious Diseases, Harvard T. H. Chan School of Public Health, Boston, USA

^4^ Human Vaccines Project, New York (NY), USA

^#^ These authors contributed equally.

^*^ Corresponding author: Julia W. Wu (wew758@mail.harvard.edu)

**SUPPLEMENTAL INFORMATION**

**Figure S1.** Study flow chart.

Illustration of the study population selection: 2,000 individuals were randomly selected from the fourth round of Rotterdam Study-I (RS-I-4) and second round of Rotterdam Study-II (RS-II-2) between 2002-2005. The criteria for inclusion included the availability of informed consent and valid plasma samples at the rounds. Data analyzed in this study included 1,930 participants with physiological and clinical information available.


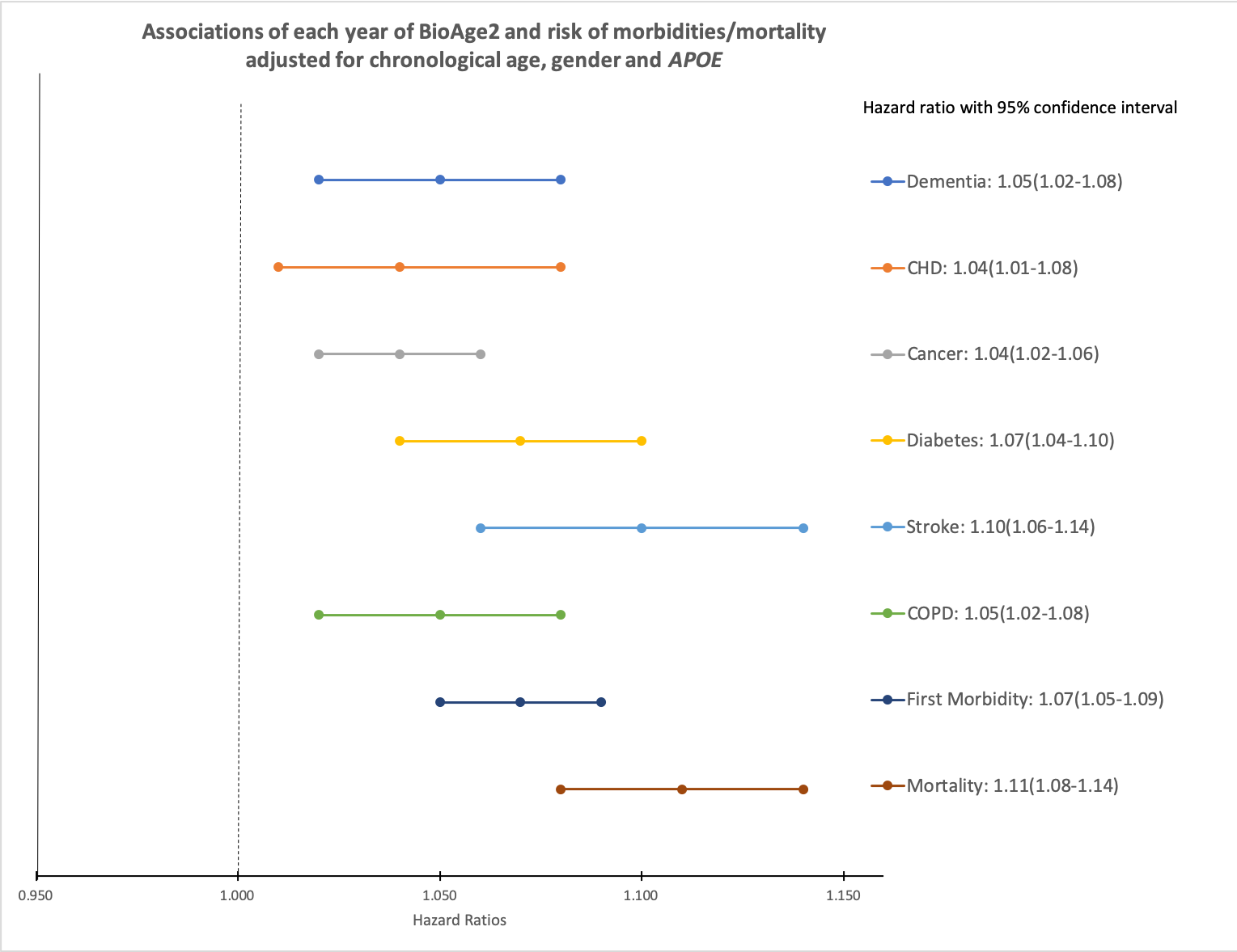


**Figure S2.** Association of BioAge2 and risk of morbidities /mortality, with adjustment for chronological age, *APOE*4 and gender, the Rotterdam Study, 2002–2012.

| **Table S1.** **Gompertz coefficients for PhenoAge, developed in the NHANES study (3).**   \| Variable \| Marker of \| Units \| Weight \| \| --- \| --- \| --- \| --- \| \| Albumin \| Liver \| g/L \| -0.0336 \| \| Creatinine \| Kidney \| umol/L \| 0.0095 \| \| Glucose, serum \| Metabolic \| mmol/L \| 0.1953 \| \| C-reactive protein (log) \| Inflammation \| mg/dL \| 0.0954 \| \| Lymphocyte percent \| Immune \| % \| -0.0120 \| \| Mean (red) cell volume \| Immune \| fL \| 0.0268 \| \| Red cell distribution width \| Immune \| % \| 0.3306 \| \| Alkaline phosphatase \| Liver \| U/L \| 0.0019 \| \| White Blood Cell count \| Immune \| 1000 cells/uL \| 0.0554 \| \| Age \|  \| Years \| 0.0804 \| \| Constant \|  \|  \| -19.9067 \| \| Gamma \|  \|  \| 0.0077 \|   **Table S2. MicroRNA aging signature according to chronological age**. The top 20 miRNAs that were most significantly associated with PhenoAge with gender adjustment, among the 591 well-expressed miRNAs in the n=1930 study population. |  |  |  |  |  |
| --- | --- | --- | --- | --- | --- | --- | --- | --- | --- | --- | --- | --- | --- | --- | --- | --- | --- | --- | --- | --- | --- | --- | --- | --- | --- | --- | --- | --- | --- | --- | --- | --- | --- | --- | --- | --- | --- | --- | --- | --- | --- | --- | --- | --- | --- | --- | --- | --- | --- | --- | --- | --- | --- | --- | --- | --- | --- |
|  |  |  |  |  |  |

| **miRNA** | **Mean Expression** | **Coefficient (Log2)** | **Fold Change** | **P-Value** | **FDR-adjusted P-Value** |
| --- | --- | --- | --- | --- | --- |
|  | **(Log2)** |  | **/40 Years** |  |  |
| miR-19b-3p | 11.17 | -0.039 | 0.34 | 4.27E-79 | 2.52E-76 |
| miR-3141 | 10.20 | 0.026 | 2.03 | 1.33E-66 | 3.93E-64 |
| miR-197-5p | 12.12 | 0.044 | 3.40 | 1.23E-62 | 2.41E-60 |
| miR-19a-3p | 9.22 | -0.023 | 0.53 | 5.64E-59 | 8.33E-57 |
| miR-93-5p | 10.38 | -0.022 | 0.54 | 9.21E-59 | 1.09E-56 |
| miR-658 | 9.85 | 0.021 | 1.81 | 1.62E-57 | 1.60E-55 |
| miR-17-3p | 8.23 | -0.020 | 0.58 | 3.22E-57 | 2.72E-55 |
| miR-6887-5p | 7.66 | 0.029 | 2.21 | 5.10E-54 | 3.76E-52 |
| miR-106b-5p | 9.75 | -0.017 | 0.63 | 1.11E-49 | 7.30E-48 |
| miR-146a-5p | 10.88 | -0.039 | 0.34 | 1.34E-49 | 7.94E-48 |
| miR-185-5p | 10.72 | -0.032 | 0.41 | 1.39E-48 | 7.47E-47 |
| miR-92a-3p | 11.14 | -0.018 | 0.60 | 5.12E-47 | 2.52E-45 |
| miR-4447 | 8.29 | 0.029 | 2.24 | 1.86E-45 | 8.44E-44 |
| miR-150-5p | 12.19 | -0.020 | 0.58 | 3.53E-45 | 1.39E-43 |
| miR-425-5p | 9.07 | -0.020 | 0.57 | 3.49E-45 | 1.39E-43 |
| miR-324-3p | 8.96 | -0.018 | 0.61 | 4.04E-45 | 1.49E-43 |
| miR-1287-5p | 12.12 | 0.031 | 2.34 | 1.46E-44 | 5.06E-43 |
| miR-6722-3p | 7.65 | 0.021 | 1.81 | 6.14E-44 | 2.02E-42 |
| miR-25-3p | 9.72 | -0.018 | 0.60 | 4.57E-43 | 1.42E-41 |
| miR-652-3p | 8.47 | -0.018 | 0.60 | 5.25E-41 | 1.55E-39 |

**Table S3. MicroRNA aging signature according to BioAge1.** The top 20 miRNAs that were most significantly associated with BioAge1 with gender adjustment, among the 591 well-expressed miRNAs in the n=1930 study population.

| **miRNA** | **Mean Expression** | **Coefficient (Log2)** | **Fold Change** | **P-Value** | **FDR-adjusted P-Value** |
| --- | --- | --- | --- | --- | --- |
|  | **(Log2)** |  | **/40 Years** |  |  |
| miR-19b-3p | 11.17 | -0.037 | 0.36 | 2.20E-112 | 1.30E-109 |
| miR-3141 | 10.19 | 0.025 | 2.00 | 4.56E-105 | 1.35E-102 |
| miR-197-5p | 12.12 | 0.040 | 3.07 | 1.29E-84 | 2.54E-82 |
| miR-93-5p | 10.39 | -0.021 | 0.56 | 1.60E-80 | 2.37E-78 |
| miR-106b-5p | 9.75 | -0.016 | 0.64 | 1.63E-77 | 1.93E-75 |
| miR-19a-3p | 9.22 | -0.021 | 0.56 | 1.28E-76 | 1.26E-74 |
| miR-6887-5p | 7.66 | 0.027 | 2.10 | 7.33E-74 | 6.19E-72 |
| miR-17-3p | 8.23 | -0.017 | 0.62 | 2.05E-68 | 1.52E-66 |
| miR-658 | 9.86 | 0.019 | 1.68 | 5.61E-68 | 3.68E-66 |
| miR-146a-5p | 10.88 | -0.036 | 0.37 | 5.64E-67 | 3.33E-65 |
| miR-6870-5p | 7.70 | 0.024 | 1.96 | 8.44E-66 | 4.53E-64 |
| miR-92a-3p | 11.14 | -0.017 | 0.62 | 1.77E-65 | 8.73E-64 |
| miR-16-5p | 12.68 | -0.017 | 0.62 | 1.67E-64 | 7.58E-63 |
| miR-25-3p | 9.72 | -0.018 | 0.61 | 1.77E-63 | 7.46E-62 |
| miR-185-5p | 10.73 | -0.029 | 0.45 | 5.72E-63 | 2.25E-61 |
| miR-1287-5p | 12.12 | 0.028 | 2.16 | 3.79E-57 | 1.40E-55 |
| miR-4681 | 6.78 | -0.024 | 0.51 | 5.61E-57 | 1.95E-55 |
| miR-6722-3p | 7.65 | 0.019 | 1.71 | 6.94E-56 | 2.28E-54 |
| miR-6810-3p | 8.89 | 0.012 | 1.39 | 1.32E-55 | 4.10E-54 |
| miR-7107-5p | 9.64 | 0.021 | 1.78 | 1.31E-54 | 3.86E-53 |

**Table S4. MicroRNA aging signature according to PhenoAge**. The top 20 miRNAs that were most significantly associated with PhenoAge with gender adjustment, among the 591 well-expressed miRNAs in the n=1930 study population.

|  |
| --- |

| **miRNA** | **Mean Expression** | **Coefficient (Log2)** | **Fold Change** | **P-Value** | **FDR-adjusted P-Value** |
| --- | --- | --- | --- | --- | --- |
|  | **(Log2)** |  | **/40 Years** |  |  |
| miR-19b-3p | 11.17 | -0.037 | 0.36 | 1.80E-109 | 1.06E-106 |
| miR-3141 | 10.19 | 0.025 | 1.98 | 3.18E-100 | 9.39E-98 |
| miR-197-5p | 12.12 | 0.040 | 3.01 | 3.67E-80 | 7.23E-78 |
| miR-93-5p | 10.39 | -0.020 | 0.57 | 2.33E-76 | 3.44E-74 |
| miR-19a-3p | 9.22 | -0.021 | 0.56 | 4.51E-75 | 5.33E-73 |
| miR-106b-5p | 9.75 | -0.016 | 0.64 | 1.39E-73 | 1.37E-71 |
| miR-6887-5p | 7.66 | 0.026 | 2.08 | 3.46E-71 | 2.92E-69 |
| miR-16-5p | 12.68 | -0.018 | 0.61 | 7.20E-67 | 5.32E-65 |
| miR-6870-5p | 7.70 | 0.024 | 1.96 | 8.64E-65 | 5.10E-63 |
| miR-92a-3p | 11.14 | -0.017 | 0.62 | 8.37E-65 | 5.10E-63 |
| miR-146a-5p | 10.88 | -0.036 | 0.37 | 3.77E-64 | 2.03E-62 |
| miR-17-3p | 8.23 | -0.017 | 0.63 | 1.31E-63 | 6.43E-62 |
| miR-658 | 9.86 | 0.018 | 1.65 | 4.54E-63 | 2.07E-61 |
| miR-25-3p | 9.72 | -0.017 | 0.62 | 1.70E-61 | 7.18E-60 |
| miR-185-5p | 10.73 | -0.028 | 0.46 | 3.08E-59 | 1.21E-57 |
| miR-4681 | 6.78 | -0.024 | 0.51 | 3.57E-55 | 1.32E-53 |
| miR-6810-3p | 8.89 | 0.012 | 1.39 | 1.34E-53 | 4.65E-52 |
| miR-4459 | 9.44 | 0.014 | 1.47 | 2.33E-53 | 7.65E-52 |
| miR-7107-5p | 9.64 | 0.021 | 1.77 | 1.46E-52 | 4.54E-51 |
| miR-6722-3p | 7.65 | 0.019 | 1.69 | 1.83E-52 | 5.41E-51 |
